# Associations between supermarket availability and body size in Australia: a cross-sectional observational study comparing state and territory capital cities

**DOI:** 10.1186/s12889-021-10458-9

**Published:** 2021-02-25

**Authors:** Suzanne J. Carroll, Gavin Turrell, Michael J. Dale, Mark Daniel

**Affiliations:** 1 Australian Geospatial Health Laboratory, Health Research Institute, University of Canberra, 23B21, 11 Kirinari St, Bruce, ACT Australia; 2 Department of Medicine, St Vincent’s Hospital, The University of Melbourne, Fitzroy, Victoria Australia

**Keywords:** Residence characteristics, Body size, BMI, Waist circumference, Supermarket availability

## Abstract

**Background:**

Residential environment features such as availability of supermarkets may shape dietary behaviour and thus overweight and obesity. This relationship may not be consistent between cities. This Australian national-level study examined: 1) the relationship between supermarket availability and body size; and 2) whether this relationship varied by capital city.

**Methods:**

This study used 2017–18 Australian National Health Survey data including individual-level socio-demographic information (age, sex, country of birth, education, occupation, household income), and measured body size (height and weight to derive body mass index [BMI], and waist circumference [WC]). Objectively-expressed measures of residential environments included: counts of supermarkets (major chain outlets), counts of amenities (representing walkable destinations including essential services, recreation, and entertainment), and area of public open space - each expressed within road-network buffers at 1000 m and 1500 m; population density (1km^2^ grid cells); and neighbourhood disadvantage (Index of Relative Socioeconomic Disadvantage) expressed within Statistical Area Level 1 units. Data for adult respondents ≥18 years residing in each of Australia’s state and territory capital cities (*n* = 9649) were used in multilevel models to estimate associations between supermarket availability and body size sequentially accounting for individual and other environment measures. An interaction term estimated city-specific differences in associations between supermarket availability and body size. Models were consequently repeated stratified by city.

**Results:**

Body size (BMI and WC) and supermarket availability varied between cities. Initial inverse associations between supermarket availability and body size (BMI and WC) were attenuated to null with inclusion of all covariates, except for BMI in the 1000 m buffer model (beta = − 0.148, 95%CI -0.27, − 0.01, *p* = 0.025). In stratified analyses, the strengths of associations varied between cities, remaining statistically significant only for some cities (BMI: Melbourne, Brisbane Hobart; WC: Brisbane, Hobart) in fully adjusted models. Different patterns of attenuation of associations with inclusion of covariates were evident for different cities.

**Conclusions:**

For Australian capital cities, greater availability of supermarkets is associated with healthful body size. Marked between-city variations in body size, supermarket availability, and relationships between supermarket availability and body size do not, however, support universal, “one-size-fits-all” solutions to change built environments to support healthful body size.

## Background

Obesity has reached epidemic proportions, particularly in developed nations [1]. Obesity arises from an energy imbalance with excess energy intake (i.e., from diet) in relation to energy usage (metabolic needs plus additional energy use through physical activity) with other factors, such as genetics, impacting on the state of this balance [2]. In response to the global prevalence of obesity international health authorities – such as the World Health Organisation – have called for governments to introduce policies that discourage the procurement of energy dense and nutritionally poor foods (e.g., fast food) and encourage the purchase and consumption of healthful foods (e.g., fruits and vegetables) [3].

Individual dietary consumption (i.e., choice of food eaten) may be influenced by a range of factors as outlined in the ecologically based conceptual model proposed by Glanz and colleagues [4]. The model identifies four types of food environments: the *community food environment* (i.e., types and locations of food stores, nature of food sold, and their hours of operation); the *organisational food environment* (e.g., food sources within the home, and school or workplace cafeterias); the *consumer environment* (within store exposures including food types and quality, prices, promotions, and placements); and the *information environment* (e.g., advertising and media reports). Each of these environments can be shaped by government and industry policies [4].

The model proposes two pathways of environmental influence, a direct pathway through the provision of access to resources, and an indirect pathway whereby environmental effects are moderated or mediated by demographic, psychosocial, or perceived environmental variables [4]. The current study focuses on the direct pathway conceiving the community food environment as an ‘enabling’ factor, a behavioural antecedent facilitating the realisation of a given motivation (e.g., eating healthful or unhealthful food) [5]. For example, living close to a supermarket may enable the purchase of fruits and vegetables supporting a healthful diet, assuming an individual is so motivated. Previous studies have reported perceived local availability of supermarkets as well as objectively measured access and availability of supermarkets or other healthful food source are related to greater fruit and vegetable intake [6–8].

To date, despite many studies having examined the relationship between the food environment and obesity, definitive evidence regarding the contribution of the community food environment to obesity remains elusive. Whilst some studies report associations between features of the food environment (e.g., availability of supermarkets or fast food) and obesity, others do not [9]. Conclusions from review articles also differ. Some reviews conclude there is fairly consistent evidence of associations between the community food environment and body size e.g. [10, 11]., However, more recent reviews conclude that, although there are patterns in the findings (e.g., greater supermarket availability is more often negatively associated with obesity than positively associated), the majority of tested associations are null e.g., [12–14]. Our understanding of the relationship between the community food environment and obesity is still at a nascent stage; in part because the evidence-base is replete with inconsistent findings, is characterised by methodological heterogeneity which makes synthesising results difficult, and is, unfortunately, of low scientific quality [12, 14, 15].

The vast majority of studies examining the relationship between the community food environment and obesity have been undertaken in the US [10, 13, 14]. However, different spatialising processes function in different countries with differences in the social, cultural, economic, and regulatory environment resulting in different spatial patterning of populations and food resource availability [16]. Food environments appear to differ between high income countries [17]. Further, whilst there is clear evidence of income-based and racial disparities in the food environment in the US, elsewhere for other high-income countries the evidence is equivocal [18]. Thus, the generalisability of findings from the US to other developed countries is questionable [10, 16, 17, 19].

A recent review of Australian food environment literature found that only a small number of studies (*n* = 13), with heterogenous methods and environmental foci, had examined the association between the community food environment and obesity [15]. The evidence was inconsistent. Two studies reported food environments were related to obesity in the expected directions (e.g., more unhealthful food environment is associated with greater BMI or obesity) [20, 21]; five studies reported the hypothesised relationships only for particular sub-groups or spatial scales [22–26]; five studies reported null findings [27–31]; and one study reported results in an unexpected direction (men and children with fast food available nearby had lower BMI than those living further from a fast food outlet) [32]. The Australian evidence-base is, therefore, like its international counterpart, inadequate as a basis for informing policy-based actions to improve the community retail environment in ways that might address the obesity epidemic.

This study aimed to improve understandings of the relationship between the community food environment and obesity in the Australian context by evaluating these associations nationally and for each capital city. Previous Australian research has had limited generalisability, being conducted in limited geographic contexts (e.g., suburbs within a single city, or a specific rural or regional area) with most work undertaken in the state of Victoria, which represents just 26% of Australia’s population [33]. We estimated the associations between supermarket availability and body size (body mass index [BMI] and waist circumference [WC]) for the capital cities of Australia’s six states and two territories undertaking both pooled and stratified analyses. The scope of our focus maximised variation in the exposure (count of supermarkets) and enabled examination of associations across areas with different food environments. Two questions were posed: 1) Is the relationship between supermarket availability and body size for major city urban environments at the national-level generalisable to the capital city level?; and 2) Are relationships between supermarket availability and body size in a given capital city generalisable to all other capital cities?

## Methods

This observational study used data from the most recent Australian National Health Survey (NHS). The 2017–18 NHS was designed and implemented by the Australian Bureau of Statistics (ABS) and, for the first time, included objectively measured residential environment characteristics representing food and physical activity (PA) environments which were constructed using a Geographic Information System. Full details of the survey’s sampling and data collection methods are documented elsewhere [34]. Only a brief overview is provided here.

### Sampling

The NHS was conducted across urban, rural, and remote areas in all six states and the two territories of Australia, over 12 months (July 2017 to June 2018). Individual dwellings (*n* = 25,109) were selected at random using a stratified multi-stage area sample of private dwellings designed to provide detailed estimates for capital cities. Out-of-scope dwellings (e.g., vacant, or derelict buildings) were excluded, yielding 21,544 sampled dwellings with 16,384 dwelling-level survey responses (76.1% response rate). For each responding dwelling, one adult (age 18 years and over), and, where appropriate, one child (0–17 years) were randomly selected, resulting in a total sample of 21,315 persons. This present study focuses on the adult sample (*n* = 16,370).

### Data collection

Trained ABS personnel collected data via face-to-face Computer Assisted Personal Interviews. Collected information pertained to long-term health conditions, disability status, mental health and psychological wellbeing, medication use, health literacy, health-related behaviours and risk factors, household demographic and socioeconomic characteristics, and household residential address.

### Geographic scope and spatial units for this study

NHS participant household addresses were geocoded enabling the spatial association with other datasets and the expression of residential environment characteristics including area-level socioeconomic indices, the food and PA environment, and population density [34]. To maintain confidentiality of NHS participant information, geocoding, spatial association and the construction of buffers and environmental measures were conducted by the ABS. Environmental characteristics were expressed for three different types of spatial units: 1000 m and 1500 m road-network distance buffers centred on NHS respondent place of residence; 1km^2^ grid cells; and ABS Statistical Area Level 1 units (SA1s) [34]. Measures expressed within buffers included: count of supermarkets; count of amenities (representing walkable destinations including essential services, recreation, and entertainment); and area of public open space (POS). Population density was expressed within 1km^2^ grid cells, and neighbourhood disadvantage was expressed within SA1s. Previous Australian studies have used similar road-network buffers [24, 25, 35]. Compared with Euclidean (straight-line) buffers, network buffers represent more plausible travel routes and contain less measurement error [36, 37]. The ABS created the network buffers using ArcGIS Network Analyst (Esri, version 9.3.1; Redlands, CA, USA). SA1s include an average of 400 individuals and are the smallest unit at which census data are generally available [38]. Analyses were conducted at the level of the individual, accounting for clustering of participants within SA1s.

### Measures

#### Supermarket availability

Supermarket availability was defined as the count of major chain supermarkets (ALDI, Coles, Foodland, Foodworks, Franklins, Fresh Market, Friendly Grocer, IGA, Safeway, and Woolworths) for the 1000 m and 1500 m road-network buffers centred on respondent place of residence. Supermarket data were sourced from HERE data (MapData Services, Sydney, Australia, 2018). Supermarket availability reflects the opportunity to purchase a wide variety of foods, including healthier options, and is generally considered to represent healthful food availability [39].

#### Body size

This study used two measures to represent body size: BMI (as weight (kg)/stature (m)^2^) and WC (cm). These physical measurements were collected from adult NHS respondents on a voluntary basis using standard procedures by trained ABS personnel. Digital scales were used to measure weight (to the nearest 100 g), a stadiometer to measure height (to the nearest mm), and a flexible metal tape to measure waist circumference (at the height midway between the lowest palpable rib and the iliac crest) [34, 40]. These measures had non-response rates of 33.8% for BMI and 35.4% for WC thus the ABS imputed missing values using the Hot Decking method [41]. The authors performed sensitivity analyses by modelling BMI and WC outcomes in a multilevel linear analysis using two-way interactions between supermarket availability and each covariate, and the imputation-identifier. In effect, these models tested whether associations between the exposure or covariates and the outcomes were different for the measured or imputed data. None of these interactions were statistically significant.

#### Covariates

Potential confounders of associations between supermarket availability and body size were identified a priori from previous literature [7, 12, 20–28, 30, 35, 42–44]. At the individual-level, these were: age (years); sex (male or female); country of birth (Australian-born or overseas-born); highest educational qualification completed (four categories: bachelor’s degree or higher, diploma, certificate, or high school or less); occupation (four categories: manager and professional, white collar employee, blue collar worker, and ‘not in the labour market’ (e.g., unemployed, retired, home duties); and total equivalised household income (as quintiles, with Q5 being the lowest category).

At the area-level, potential confounders were: neighbourhood disadvantage (expressed as a continuous variable for SA1s using the ABS Index of Relative Socioeconomic Disadvantage (IRSD) [45] with lower scores denoting more disadvantaged areas); population density (persons per 1km^2^ grid-cell sourced from the Australian Population Grid 2017 [46]); and the PA environment expressed two ways for 1000 m and 1500 m network buffers: 1) count of local amenities that residents might theoretically access by walking or active transport (i.e., essential services [e.g., banks, post offices, medical services, pharmacies], education and community [e.g., schools, libraries, community centres, places of worship], hospitality, entertainment, retail [excluding supermarkets], and recreational facilities [e.g., sports centres]); and 2) area of POS (sum of POS parcels greater than 1 ha, including but not limited to sporting fields, playgrounds, picnic areas, parks, gardens, and other open spaces). Due to the distribution of the POS area measure, quintiles were used in analysis models. Amenities data were sourced from the HERE Place-of-Interest data layer (MapData Services, Sydney, Australia, 2018). POS data were sourced from the Transport and Topography dataset [47].

### Analysis

The primary pool of *n* = 16,370 adult NHS participants was restricted to adult residents of capital cities (*n* = 9881). Respondents self-reporting as pregnant or with missing data for the exposures, covariates or outcomes were excluded, yielding an analytic sample of *n* = 9649 persons.

**Fig. 1:**
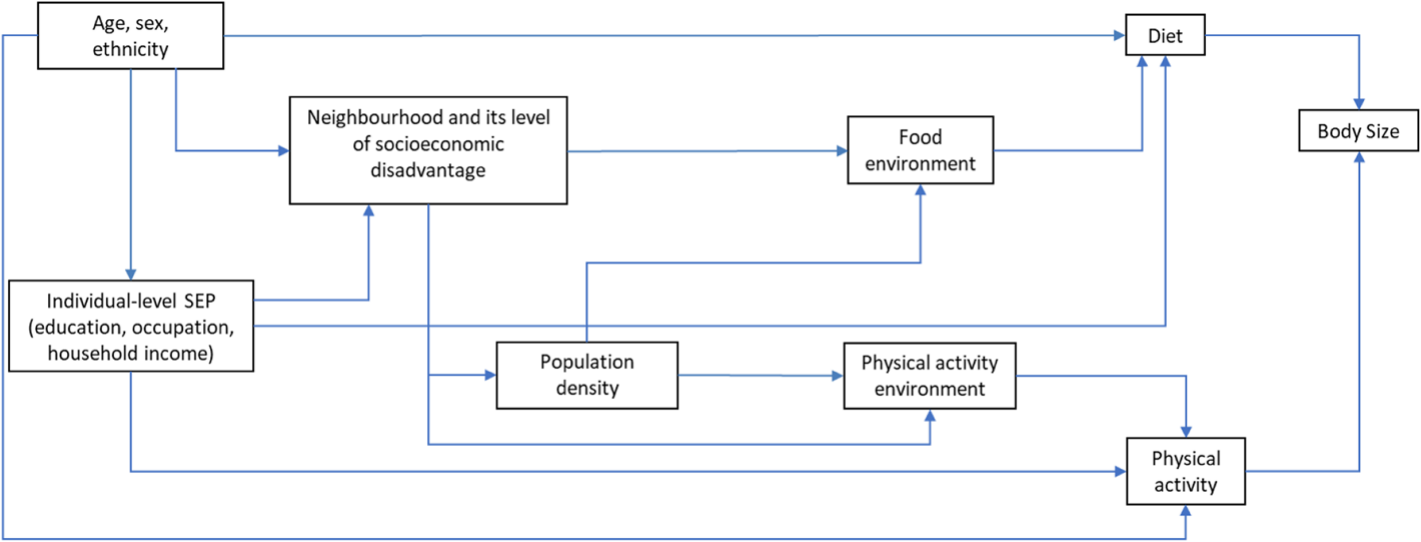
Directed Acyclic Graph depicting relationships between neighbourhood supermarkets and body size (BMI and WC)

To guide analysis, a Direct Acyclic Graph (DAG) was constructed (Fig. 1) based on the current literature (e.g., [7, 12, 20–28, 30, 35, 42–44]). Individual-level demographic and socioeconomic factors were conceptualised as influencing the probability of self-selection into neighbourhoods that vary in their level of disadvantage, and as predictors of diet (energy intake) and PA (energy expenditure). Although diet and PA are depicted in the DAG as being proximal causes of body size (i.e., mediators between environmental features and body size), diet and PA were not included in analytic models due to the likelihood of over adjustment. Diet and PA behaviours are represented for completeness and to demonstrate the biological plausibility of the DAG. At the area-level, the DAG posits that neighbourhoods within cities differ in levels of socioeconomic disadvantage and population density, and that these factors are likely to be associated with city-differences in their food and PA environments. Moreover, within each city, the food and PA environments are correlated (e.g., in the NHS data, the city-combined correlations between supermarkets and count of amenities at 1000 m and 1500 m are rho = 0.64 and rho = 0.73 respectively), hence associations between the PA environment and body size might confound associations between supermarket availability and BMI and WC.

Multilevel linear regression models were used to directly test the interaction between supermarket availability and cities (as categories) in relation to body size at 1000 m and 1500 m, accounting for the clustering of individuals within neighbourhoods (SA1s). In accordance with the DAG, a five-stage modelling strategy was used: Model 1) supermarket availability, city, their interaction term (supermarket availability * city) adjusted for age and sex; Model 2) model 1 plus country of birth and neighbourhood disadvantage; Model 3) model 2 plus individual-level socioeconomic position (SEP; education, occupation, and household income); Model 4) model 3 plus the PA environment (count of amenities and POS area); and Model 5) model 4 plus population density. The interaction term (supermarket availability * city) was statistically significant at the 1000 m buffer size (BMI: *p* = 0.040; WC: *p* = 0.007) but not the 1500 m buffer size (BMI: *p* = 0.107; WC: *p* = 0.168) suggesting differences in associations between supermarket availability and body size according to city. Given the statistically significant interaction effect at the 1000 m buffer size, the above modelling approach was repeated (for both buffer sizes), stratified by city. Only the results of the stratified models are presented here. Due to some moderate to high correlations (as noted above) we assessed potential collinearity issues within models by calculating variance inflation factors (VIFs). The maximum VIF was 2.7 (for amenities in the 1500 m buffer model) indicating some collinearity but not at a concerning level. All data preparation and analyses were conducted within the ABS DataLab using Stata 16 (StataCorp. 2017. *Stata Statistical Software: Release 16*. College Station, TX, USA) with statistical significance set at alpha = 0.05.

## Results

Table 1 presents descriptive statistics for BMI, WC, supermarket availability, and the covariates, for each capital city and all cities combined. Mean BMI ranged from 27.4 kg/m^2^ in Sydney, to 28.5 kg/m^2^ in Brisbane; and mean WC ranged from 91.5 cm in Darwin, to 94.3 cm in Adelaide. Supermarket availability varied widely between cities, from Hobart (mean availability of 0.3 supermarkets within 1000 m and 0.69 within 1500 m of respondents’ residences), to Sydney (0.93 within 1000 m and 1.91 within 1500 m). There were substantial sociodemographic differences between cities in terms of age, country of birth, and individual-level SEP. Neighbourhoods in Canberra were the most socioeconomically advantaged and neighbourhoods in Hobart the most disadvantaged. Counts of amenities, POS area, and population density each showed marked variation between the capital cities.

**Table 1:** Characteristics of study participants and their environments, by city

	Sydney ***N*** = 1744	Melbourne ***N*** = 1782	Brisbane ***N*** = 1416	Adelaide ***N*** = 1111	Perth ***N*** = 1222	Hobart ***N*** = 547	Darwin ***N*** = 734	Canberra ***N*** = 1093	All cities ***N*** = 9649
***Body size***									
Body mass index (kg/m^2^)									
Mean (SD)	27.4 (5.7)	27.7 (5.4)	28.5 (6.0)	28.4 (5.8)	27.9 (5.7)	27.7 (5.7)	27.6 (5.8)	27.8 (5.9)	27.9 (5.8)
*10th / 90th percentile*	21.2 / 34.6	21.5 / 34.7	21.4 / 36.4	21.8 / 36.3	21.6 / 35.5	21.0 / 34.9	21.2 / 35.1	21.6 / 35.4	21.5 / 55.4
Waist circumference (cm)									
Mean (SD)	91.8 (15.5)	92.2 (15.0)	94.1 (16.2)	94.3 (15.8)	92.4 (15.0)	91.6 (14.9)	91.5 (16.6)	92.7 (15.6)	92.7 (15.6)
*10th / 90th percentile*	73 / 112	73 / 111	74 / 116	75 / 114	74 / 112	73 / 110	72 / 112	74 / 112	73 / 112
***Food environment***									
Count of supermarkets, 1000 m									
Mean (SD)	0.93 (1.3)	0.86 (1.4)	0.63 (1.0)	0.76 (1.1)	0.55 (0.8)	0.30 (0.6)	0.43 (0.9)	0.39 (0.7)	0.67 (1.1)
*10th / 90th percentile*	0 / 3	0 / 2	0 / 2	0 / 2	0 / 2	0 / 1	0 / 2	0 / 1	0 / 2
Range	0–9	0–13	0–7	0–7	0–4	0–2	0–5	0–4	0–13
Count of supermarkets, 1500 m									
Mean (SD)	1.91 (2.0)	1.94 (2.5)	1.38 (1.7)	1.74 (1.7)	1.15 (1.2)	0.69 (0.9)	0.80 (1.3)	0.93 (1.1)	1.46 (1.8)
*10th / 90th percentile*	0 / 5	0 / 5	0 / 3	0 / 4	0 / 3	0 / 2	0 / 3	0 / 2	0 / 4
Range	0–15	0–22	0–12	0–11	0–6	0–3	0–5	0–5	0–22
***Covariates***									
Age (mean, SD)	49.9 (18.1)	48.9 (18.5)	48.1 (17.9)	51.7 (18.4)	49.5 (18.3)	52.1 (18.2)	45.0 (16.0)	48.5 (17.7)	49.2 (18.1)
Sex (% men)	45.4	47.1	45.5	45.9	46.1	40.2	49.9	42.8	45.6
*Country of birth (%)*									
Australia	49.4	57.5	68.9	68.9	54.3	80.6	63.5	67.3	61.5
Overseas	50.6	42.5	31.1	31.1	45.7	19.4	36.5	32.7	38.5
*Education (%)*									
Bachelor’s degree+	37.2	37.2	29.2	26.2	28.6	31.6	30.5	44.3	33.6
Diploma	10.7	11.7	12.6	10.2	12.1	10.4	10.1	11.0	11.2
Certificate	14.9	14.2	21.5	18.8	19.1	21.4	23.8	12.3	17.5
High school or less	33.3	34.3	34.3	40.9	36.3	34.0	33.4	28.6	34.4
Missing	4.0	2.6	2.3	4.0	4.0	2.6	2.2	3.9	3.3
*Occupation (%)*									
Managers and professionals	28.1	28.6	23.7	24.8	24.0	23.2	23.7	36.8	27.0
White collar	18.8	20.2	22.1	19.0	17.9	20.3	27.5	21.5	20.5
Blue collar	14.1	14.7	18.2	14.6	19.1	13.9	22.2	10.4	15.7
Not in labour market	39.0	36.5	36.0	41.7	39.0	42.6	26.6	31.3	36.8
Household income (quintiles) *(%)*									
Q1 (highest)	16.0	13.9	17.0	22.3	18.7	22.5	14.0	11.0	16.5
Q2	13.3	16.4	15.9	19.7	13.8	20.5	12.3	12.3	15.3
Q3	15.3	16.7	18.4	17.8	16.9	18.1	17.7	15.8	16.9
Q4	16.6	18.0	16.3	16.9	17.4	16.6	19.1	19.9	17.5
Q5 (lowest)	22.9	15.8	17.5	14.7	16.8	14.1	25.5	29.6	19.5
Missing	15.8	19..3	14.9	8.6	16.5	8.2	11.4	11.5	14.3
*Area-level disadvantage*									
Mean (SD)	1019.9 (109.0)	1021.4 (87.8)	1011.8 (90.5)	992.8 (92.6)	1022.2 (75.3)	987.2 (103.8)	1029.2 (76.9)	1066.0 (53.4)	1020.2 (90.9)
*10th / 90th percentile*	883 / 1120	908 / 1106	894 / 1108	859 / 1089	919 / 1107	835 / 1095	955 / 1110	998 / 1127	899 / 1111
Range	325–1165	506–1155	486–1159	573–1140	563–1152	540–1134	720–1146	809–1165	325–1165
Count of amenities, 1000 m									
Mean (SD)	112.1 (249.0)	88.4 (246.5)	38.7 (82.3)	47.4 (76.4)	25.4 (44.8)	38.0 (71.6)	27.4 (48.6)	20.2 (58.1)	57.5 (162.3)
*10th / 90th percentile*	5 / 300	4 / 202	1 / 91	4 / 117	1 / 59	1 / 107	2 / 55	0 / 35	2 / 136
Range	0–2882	0–3717	0–1001	0–1053	0–589	0–500	0–241	0–659	0–3717
Count of amenities, 1500 m									
Mean (SD)	232.6 (447.8)	207.1 (499.0)	84.9 (176.8)	109.9 (172.3)	60.1 (87.6)	90.2 (157.7)	55.5 (72.1)	50.5 (107.6)	128.1 (315.4)
*10th / 90th percentile*	14 / 558	13 / 450	4 / 196	12 / 244	4 / 130	3 / 211	7 / 211	2 / 95	7 / 291
Range	0–5301	0–6091	0–2175	0–1847	0–1011	0–922	0–288	0–759	0–6091
Area of public open space, 1000 m^a^									
Mean (SD)	146.8 (1074.2)	72.3 (176.1)	92.0 (1289.1)	3636.0 (35,995.0)	743.4 (3793.0)	45,925.1 (293,905.1)	130.4 (405.2)	863.8 (16,811.7)	3277.4 (71,988.1)
*10th / 90th percentile*	0 / 94	4 / 167	1 / 69	2 / 89	9 / 501	2 / 202	1 / 119	16 / 303	2 / 176
Range	0–15,744	0–2714	0–45,054	0–363,544	0–37,903	0–1,927,958	0–1685	0–393,282	0–1,927,958
Area of public open space, 1500 m^a^									
Mean (SD)	339.4 (1804.8)	147.5 (247.6)	223.1 (2439.8)	4333.9 (39,095.8)	1125.6 (4118.2)	112,989.9 (452,817.2)	306.4 (585.0)	4243.2 (39,249.3)	7672.2 (112,370.1)
*10th / 90th percentile*	8 / 196	20 / 322	9 / 126	9 / 190	29 / 2610	12 / 1563	8 / 1658	52 / 578	12 / 414
Range	0–17,989	1–2718	0–45,265	0–363,553	0–38,088	0–1,927,958	0–1702	6–393,339	0–1,927,958
Population density									
Mean (SD)	3958.6 (2916.6)	3008.4 (2270.3)	1998.3 (1173.5)	2055.6 (727.7)	1764.9 (945.9)	1244.3 (848.7)	1482.4 (778.4)	1682.9 (807.2)	2398.5 (1954.2)
*10th / 90th percentile*	1071 / 7976	1017 / 4783	478 / 3361	969 / 2839	496 / 3050	152 / 2362	275 / 2597	735 / 2747	639 / 4163
Range	0–20,384	0–25,743	1.6–7542	2.8–3622	0–4519	3.5–3084	0–2849	0–4893	0–257,436

^a^For analysis, area of public open space was categorised into quintiles

### Supermarket availability and BMI

Table 2 shows the association between supermarket availability and BMI by capital city (i.e., results of the stratified analyses). For all capital cities combined, there was a negative association between supermarket availability and BMI for both 1000 m and 1500 m buffers: individuals living in areas with greater supermarket availability had lower BMIs. This association was noticeably stronger in 1000 m buffers compared with 1500 m buffers. The relationship between supermarkets and BMI within 1000 m and 1500 m buffers was largely unaffected by adjustment for demographic factors, individual-level SEP, and neighbourhood disadvantage (Models 1–3); however, associations became non-significant on inclusion of the PA environment (count of amenities and POS area, Model 4).

**Table 2:** Supermarket availability and BMI, by capital city: linear regression coefficients and 95% confidence intervals

City	Buffer	Model 1		Model 2		Model 3		Model 4		Model 5	
		β	95%CI	β	95%CI	β	95%CI	β	95%CI	β	95%CI
All cities	1000 m	**−0.276**	− 0.37, − 0.17***	**−0.293**	− 0.39, − 0.18***	**−0.249**	− 0.35, − 0.14***	**−0.167**	− 0.29, − 0.03*	**−0.148**	− 0.27, − 0.01*
AIC	1000 m	61,051.9	–	60,880.1	–	60,810.9	–	60,810.4	–	60,804.3	–
	1500 m	**−0.162**	−0.22, − 0.10***	**−0.173**	− 0.23, − 0.11***	**−0.147**	− 0.20, − 0.08***	**−0.089**	− 0.17, − 0.00*	−0.076	− 0.16, 0.10
AIC	1500 m	61,052.1	–	60,880.1	–	60,811.8	–	60,811.8	–	60,806.0	–
Sydney	1000 m	**−0.266**	− 0.46, − 0.06**	**−0.287**	− 0.49, − 0.08**	**−0.266**	− 0.47, − 0.06*	− 0.077	− 0.36, 0.20	− 0.026	− 0.31, 0.25
	1500 m	**−0.208**	− 0.34, − 0.07**	**−0.220**	− 0.36, − 0.08**	**− 0.207**	− 0.34, − 0.06**	−0.127	− 0.34, 0.08	− 0.079	− 0.28, 0.13
Melbourne	1000 m	**− 0.290**	− 0.49, − 0.08**	**−0.311**	− 0.51, − 0.10**	**−0.250**	− 0.45, − 0.05*	**−0.346**	− 0.60, − 0.08**	**−0.337**	− 0.59, − 0.07*
	1500 m	**− 0.151**	− 0.26, − 0.04**	**−0.159**	− 0.26, − 0.05**	**−0.128**	− 0.23, − 0.02*	**−0.223**	− 0.39, − 0.05*	**−0.220**	− 0.39, − 0.04*
Brisbane	1000 m	**− 0.585**	−0.90, − 0.26***	**−0.591**	− 0.91, − 0.26***	**−0.573**	− 0.89, − 0.25***	**−0.502**	− 0.91, − 0.08*	**−0.503**	− 0.91, − 0.08*
	1500 m	**−0.310**	− 0.47, − 0.14***	**− 0.325**	− 0.48, − 0.16***	**− 0.314**	− 0.47, − 0.15***	**−0.337**	− 0.59, − 0.08**	**−0.337**	− 0.59, − 0.08**
Adelaide	1000 m	− 0.173	− 0.43, 0.09	− 0.163	− 0.42, 0.09	− 0.065	− 0.32, 0.19	− 0.011	− 0.31, 0.28	0.007	−0.29, 0.30
	1500 m	**−0.228**	−0.39, − 0.06**	**− 0.231**	− 0.39, − 0.07*	**− 0.186**	− 0.34, − 0.02*	−0.174	− 0.36, 0.01	−0.162	− 0.34, 0.02
Perth	1000 m	0.173	− 0.20, 0.55	0.019	−0.36, 0.40	0.068	− 0.31, 0.45	0.236	− 0.20, 0.67	0.275	− 0.16, 0.71
	1500 m	0.106	− 0.16, 0.37	− 0.023	− 0.29, 0.24	0.014	− 0.26, 0.29	0.206	− 0.11, 0.52	0.257	− 0.07, 0.58
Hobart	1000 m	**− 1.08**	− 1.73, − 0.44**	**− 1.04**	− 1.65, − 0.42**	**− 0.918**	− 1.56, − 0.26**	**−1.00**	− 1.80, − 0.20*	**−0.923**	−1.73, − 0.11*
	1500 m	− 0.163	− 0.64, 0.32	− 0.089	−0.54, 0.36	0.036	− 0.41, 0.48	0.034	−0.59, 0.66	0.174	− 0.44, 0.79
Darwin	1000 m	0.004	−0.42, 0.43	0.110	−0.32, 0.54	0.153	−0.26, 0.57	− 0.491	− 1.24, 0.26	− 0.499	− 1.26, 0.26
	1500 m	0.104	−0.21, 0.42	0.160	−0.16, 0.48	0.182	−0.11, 0.48	0.270	− 0.53, 1.07	0.271	−0.53, 1.07
Canberra	1000 m	−0.281	−0.81, 0.25	− 0.329	− 0.85, 0.19	− 0.342	− 0.87, 0.18	− 0.188	− 0.87, 0.50	− 0.190	− 0.88, 0.50
	1500 m	−0.023	− 0.31, 0.26	− 0.044	−0.33, 0.24	− 0.035	− 0.31, 0.24	0.151	− 0.20, 0.51	0.150	− 0.20, 0.50

Model 1: Supermarket availability and BMI adjusted for age and sex; Model 2: Model 1 plus adjustment for country of birth and neighbourhood disadvantage; Model 3: Model 2 plus adjustment for individual-level socioeconomic position (education, occupation, and household income); Model 4: Model 3 plus adjustment for physical activity environment (walkable amenities, public open space); Model 5: Model 4 plus adjustment for population density

*p < 0.05; **p < 0.01; ***p < 0.001

At the city-level, negative associations between supermarkets and BMI were found for Sydney, Melbourne, Brisbane, Adelaide (1500 m only), and Hobart (1000 m only): greater supermarket availability was associated with lower average BMI. There were no statistically significant associations between supermarket availability and BMI in Perth, Darwin, or Canberra in any models.

In Sydney and Adelaide (1500 m buffer), a similar pattern was seen to that for the All Cities models, with attenuation of associations to non-significance on inclusion of the PA environment. In Melbourne, Brisbane, and Hobart (1000 m buffers), associations between supermarket availability and BMI were observed for all models, and the strengths of the associations were little affected by adjustment for the covariates. For cities with significant associations between supermarkets and BMI the effect-sizes were largest for Hobart (1000 m buffers), intermediate for Brisbane, and smallest for Sydney and Melbourne. It is notable that the effect-sizes in Hobart (1000 m buffers), and Brisbane (1000 m and 1500 m buffers) were between two and five-times larger than that observed for all cities combined.

### Supermarket availability and waist circumference

Table 3 presents the associations between supermarket availability and WC by city. For all cities combined, there was a significant negative association between supermarket availability and WC at both buffer sizes after adjustment for demographic factors, individual-level SEP, and neighbourhood disadvantage: individuals residing in areas with greater availability of supermarkets had, on average, smaller WC. This association was strongest using 1000 m buffers. These associations were attenuated to non-significance after adjustment for the PA environment (Model 4).

**Table 3:** Supermarket availability and WC, by capital city: linear regression coefficients and 95% confidence intervals

City	Buffer	Model 1		Model 2		Model 3		Model 4		Model 5	
		β	95%CI	β	95%CI	β	95%CI	β	95%CI	β	95%CI
All cities	1000 m	**−0.591**	−0.86, −0.31***	**−0.628**	− 0.90, − 0.35***	**−0.537**	− 0.81, − 0.26***	−0.318	− 0.64, 0.00	−0.278	− 0.60, 0.04
AIC	1000 m	78,581.4	–	78,424.6	–	78,359.8	–	78,358.6	–	78,355.4	–
	1500 m	**−0.363**	−0.54, − 0.18***	**−0.385**	− 0.56, − 0.20***	**−0.334**	− 0.51, − 0.15***	−0.187	− 0.40, 0.03	−0.158	− 0.37, 0.06
AIC	1500 m	78,580.0	–	78,423.0	–	78,358.2	–	78,358.2	–	78,355.8	–
Sydney	1000 m	−0.411	−0.88, 0.06	−0.467	− 0.95, 0.02	−0.447	− 0.93, 0.03	−0.072	− 0.36, 0.20	−0.060	− 0.57, 0.70
	1500 m	**−0.449**	− 0.77, − 0.12**	**−0.484**	− 0.82, − 0.14**	**−0.469**	− 0.80, − 0.13**	−0.362	− 0.85, 0.13	−0.256	− 0.75, 0.24
Melbourne	1000 m	**−0.673**	−1.28, − 0.05*	**−0.738**	− 1.34, − 0.13*	−0.589	−1.18, 0.00	− 0.585	−1.31, 0.14	−0.559	− 1.28, 0.16
	1500 m	−0.351	− 0.73, 0.03	**− 0.379**	−0.75, − 0.01*	−0.314	− 0.68, 0.05	−0.419	− 0.90, 0.07	−0.407	− 0.89, 0.08
Brisbane	1000 m	**−1.379**	−2.18, −0.57**	**−1.404**	− 2.19, − 0.61***	**−1.333**	−2.09, − 0.56**	**−1.300**	−2.28, − 0.31*	**−1.304**	−2.28, − 0.31**
	1500 m	**−0.682**	− 1.12, − 0.23**	**−0.726**	−1.16, − 0.28***	**−0.683**	−1.12, − 0.23**	**−0.865**	−1.52, − 0.20*	**−0.865**	−1.52, − 0.20*
Adelaide	1000 m	−0.190	− 0.89, 0.51	−0.143	− 0.84, 0.55	0.136	−0.54, 0.82	0.397	− 0.39, 1.18	0.415	−0.37, 1.20
	1500 m	**−0.533**	−0.95, − 0.10*	**−0.543**	− 0.96, − 0.12*	**−0.425**	− 0.84, − 0.00*	−0.326	− 0.80, 0.15	−0.340	− 0.82, 0.14
Perth	1000 m	0.086	−0.83, 1.01	−0.249	− 1.17, 0.67	− 0.195	−1.13, 0.73	0.296	−0.74, 1.33	0.460	− 0.59, 1.51
	1500 m	0.089	−0.54, 0.72	− 0.197	−0.85, 0.45	− 0.139	−0.81, 0.53	0.382	− 0.37, 1.13	0.550	−0.20, 1.31
Hobart	1000 m	**−2.77**	−4.27, − 1.27***	**−2.59**	−4.07, − 1.11**	**−2.34**	− 3.89, − 0.79**	**−2.35**	−4.19, − 0.52*	**−2.12**	−4.00, − 0.24*
	1500 m	−0.378	− 1.52, 0.76	− 0.169	−1.26, 0.93	0.159	−0.92, 1.24	0.140	− 1.34, 1.62	0.599	− 0.86, 2.06
Darwin	1000 m	0.252	−0.77, 1.28	0.451	−0.59, 1.50	0.558	−0.46, 1.57	−1.18	−3.50, 1.12	−1.25	−3.62, 1.11
	1500 m	0.558	−0.36, 1.47	0.668	−0.24, 1.58	0.712	−0.14, 1.56	1.35	−0.84, 3.54	1.35	−0.84, 3.55
Canberra	1000 m	−1.04	−2.18, 0.08	**−1.19**	−2.32, −0.06*	**−1.32**	−2.47, − 0.17*	−1.33	−2.81, 0.15	− 1.32	−2.81, 0.16
	1500 m	− 0.157	− 0.82, 0.50	−0.218	− 0.87, 0.43	−0.257	− 0.92, 0.40	−0.045	− 0.87, 0.78	−0.043	− 0.86, 0.76

Model 1: Supermarket availability and WC adjusted for age and sex; Model 2: Model 1 plus adjustment for country of birth and neighbourhood socioeconomic disadvantage; Model 3: Model 2 plus adjustment for individual-level socioeconomic position (education, occupation, and household income); Model 4: Model 3 plus adjustment for physical activity environment (walkable amenities, public open space); Model 5: Model 4 plus adjustment for population density

*p < 0.05; **p < 0.01; ***p < 0.001

At the city-level, significant negative associations between supermarkets and WC were observed for Sydney (1500 m only), Melbourne, Brisbane, Adelaide (1500 m only), Hobart (1000 m), and Canberra (1000 m, Models 2 and 3 only). There was no association between supermarket availability and WC in Darwin and Perth for any buffer size.

In Brisbane and Hobart (1000 m buffer), the negative association between supermarket availability and WC was largely unaffected by adjustment for the covariates. The association was stronger for the Brisbane 1000 m buffer than the 1500 m buffer. In Sydney (1500 m) and Adelaide (1500 m), the negative association became non-significant on inclusion of the PA environment, showing a similar pattern to that for the model series assessing cities overall. In Melbourne and Canberra, greater supermarket availability was associated with smaller WC; however, the statistical significance of these associations was sensitive to covariate adjustment and no clear pattern was evident. Across the cities, the strength of association between supermarket availability and WC was greatest in Hobart (1000 m) and Brisbane (1000 m), and both were substantially larger than that found for all cities combined.

## Discussion

This national study found greater residential availability of supermarkets was inversely related to individual body size (BMI and WC). This study also found that both supermarket availability, and the strength of association between supermarket availability and body size, varied according to capital city. The relationship between supermarket availability and body size for pooled Australian capital cities is not generalisable to individual capital cities. Moreover, relationships between supermarket availability and body size in one capital city do not appear generalisable to other capital cities. These findings challenge the widely assumed notion, evident in much health and place research, that study findings in a given context are generalisable to other cities in that country (or even other countries). These findings also cast doubt on the suitability of universal, “one-size-fits-all” policy solutions intended to change the built environment to reduce our rising rates of overweight and obesity. As the first national-level study of these relationships in urban Australia, our results account for the mixed findings of single-setting Australian studies of the food environment and health outcomes [15].

Large between-city variation in the availability of supermarkets was evident in this study, with lower counts of supermarkets in both 1000 m and 1500 m buffers in Hobart, Darwin and Canberra, and higher counts of supermarkets within buffers for Sydney and Melbourne. This variation likely reflects city size and population density, Sydney and Melbourne being larger cities with greater population density than Hobart, Darwin, and Canberra. It was recently reported that supermarket availability declines from the inner city to outer fringe areas of Melbourne [24, 48]. Neighbourhood disadvantage, POS area, and count of amenities also varied between capital cities, but only count of amenities was related to supermarket availability (correlation of 0.73 at the 1500 m buffer). The relationship between count of amenities and supermarket availability may underpin the attenuation of the association between supermarket availability and body size apparent for some cities on inclusion of amenities in analytic models (Model 4). A relationship between count of amenities and count of supermarkets is unsurprising as both are likely driven by population density and tend to co-locate. The implication of our results is that variation in supermarket availability and other environmental exposure measures *between* capital cities (as well as within cities), and covariation between environmental exposures (neighbourhood disadvantage and supermarket availability), indicate a limited generalisability for food environment and body size relationships *between cities within the same country* as well as, possibly, a limited generalisability between countries.

This study found, overall for Australian capital cities that living in an area with greater supermarket availability was associated with lesser body size, although this effect was attenuated with inclusion of other environmental measures, remaining statistically significant only for BMI at the 1000 m buffer. Importantly, the relationship between supermarket availability and body size varied according to capital city, with no associations in some cities for any models or buffer size (e.g., Perth and Darwin) but associations robust to inclusion of other environmental factors in other cities (e.g., both BMI and WC models for Brisbane). Whilst only 13 Australian studies [15] have assessed the food environment in relation to body size, few of these specifically assessed supermarket availability. Of studies that did, the majority were conducted in Melbourne and their findings are mixed. For example, one study reported supermarket density and proximity were not associated with BMI in established or urban growth areas [23]. Conversely, in a different Melbourne study of women only, living closer to a supermarket was associated with lower BMI, and greater density of supermarkets was associated with lesser BMI amongst more educated women [22]. Similarly, a cross-sectional study set in Melbourne reported greater supermarket availability was associated with lesser BMI for residents in high disadvantaged areas but not for those in mid or low disadvantaged areas [24].

Studies conducted in other Australian cities also present inconsistent relationships between the food environment and body size [15]. Different operationalisations of the food environment may explain some inconsistencies in findings between studies. However, the findings of the current study indicate that associations between the food environment and body size vary according to city. To our knowledge, no study has previously assessed the within-nation variation in the association between supermarket availability and body size by state or provincial capital cities, making this study an important contribution to the literature.

Between-city variation in body size and environmental characteristics, and variation in associations between body size and supermarket availability align with calls to conceive, and to research as such, environmental features and health outcomes as components of complex systems, systems which also incorporate demographic, psychosocial, and behavioural factors [49, 50]. This is supported by the different patterns of attenuation of association between supermarket availability and body size on inclusion of other environmental factors for the different cities. While city-specific variations indicate city-specific systems, our understandings and capacity to research the nuance of regionally specific systems are in their infancy. Hence researchers and policy makers should not inappropriately aggregate, conceptually or in practice, studies lacking demonstrably similar environments. Broad public health approaches assuming a one-size-fits-all approach are unlikely to elicit intended health improvements in all areas, cities, or regions. Efforts to improve health outcomes will need to be tailored according to baseline levels of area factors (environmental and prevalence of disease or risk) and area-specific relationships linking environments to health. It is important to advance research theory and practice, that is, methodology, in ways to further develop our understandings of these complex systems, notably why and how relationships may vary between cities.

Overall, our findings support the need to improve the availability of supermarkets to support healthy body size; however, the effectiveness of this strategy will vary by city. This study also demonstrates the utility of the addition of objectively expressed residential environmental exposure information now included in the Australian NHS. The addition of such measures facilitates broad-scale research into how residential environments are related to a range of individual-level health-related behaviours, risk factors, and health outcomes. This report showcases the important public health utility of routinely collected health monitoring surveys that incorporate geospatial data as part of their design. Other routinely collected datasets might similarly be improved for use in place-health research, the findings from which can inform local health and urban planning policy. Future research enabled by inclusion of geospatial measures within such datasets includes the ability to test interactions between environmental measures or between environmental and individual-level measures, investigations of the mechanisms of relationships (for example health behaviours such as diet and physical activity), and assessment of other health outcomes including potential disease sequalae such as type 2 diabetes and cardiovascular disease. Whilst our findings are important in the Australian context, they also raise questions regarding the generalisability of similar studies in other countries. It is likely that regional heterogeneity in associations exists more broadly than exclusively within Australia. Replicability of intervention success when an intervention is divorced from its original environment is uncertain where such heterogeneity is present.

Strengths of this study include the use of nation-wide data enabling estimation of associations between objectively expressed residential supermarket availability and body size for all Australian capital cities. A comprehensive set of other environmental measures was also objectively expressed and included in analytic models to account for potential confounders. Objective environmental measures are arguably preferred vis-à-vis self-reported perceived environmental measures which are influenced by individual factors [51] and can result in “same source” bias [52]. However, the accuracy of objectively expressed environmental measures in reflecting local environmental exposure levels is dependent on the accuracy of the base data layers used. Discrepancies between the constructed measures and actual exposures are likely to attenuate associations with body size towards the null. Environmental exposure measures were expressed within road-network buffers of two different distances designed to reflect reasonable walking distances, enable comparability with other studies, and to detect distance-related nuances to tested associations. However, we recognise that residents may not shop within these buffer distances [24] and that individuals may choose to purchase goods elsewhere for reasons including convenience whilst transiting to or from other tasks (e.g., work, recreation) [53].

The outcome measures, BMI and WC, were measured rather than self-reported. Most previous studies in Australia, and elsewhere, relied on self-reported body size which is influenced by social desirability bias [54]. Where individuals did not consent to being measured, body size data were imputed based on self-reported body size and other information. The impact of the use of imputed body size data was assessed using sensitivity analyses. Individual-level sociodemographic data were self-reported and therefore subject to potential self-report biases including social desirability. This study focused on availability of supermarkets although other factors may impact local food purchasing behaviours, such as within store food availability, quality, and pricing [15]. Similarly, the availability of food sources other than supermarkets is likely to impact on food purchase behaviour [55]. Supermarkets included only major chain outlets and not smaller independent outlets. Lastly, this study is cross-sectional in design and therefore causal relationship cannot be inferred.

## Conclusion

This study found that a greater availability of supermarkets was associated with more healthful body size in Australian capital cities. Underlying this main finding, and most importantly, this study further found between-city variation in supermarket availability, and variation in the association between supermarket availability and body size. This latter finding suggests as questionable the suitability of universal, “one-size-fits-all” policy solutions to change built environments to support healthful body weight, as implied by the first finding. Rather, targeted intervention strategies that account for on-the-ground context and, as much as possible, complexity of local environment-health systems, are implicated as fundamentally necessary to enable and support healthful body weights.

## Data Availability

The data that support the findings of this study are available from the Australian Bureau of Statistics. Restrictions may apply to the availability of these data, which may require applicants to undertake ABS directed training to ensure the maintenance of confidentiality. Application may be made via: www.abs.gov.au/websitedbs/D3310114.nsf/home/How+to+Apply+for+Microdata.

## Abbreviations

ABS: Australian Bureau of Statistics
Body size: BMI and WC
BMI: Body mass index
DAG: Direct Acyclic Graph
IRSD: Index of Relative Socioeconomic Disadvantage
NHS: National Health Survey
PA: Physical activity
POS: Public open space
SA1: ABS Statistical Area Level 1
SEP: Socioeconomic position
WC: Waist circumference
